# Iron-enriched *Aspergillus oryzae* as an alternative to iron sulphate to limit iron accumulation, growth and motility of the enteric pathogen *S.* Typhimurium

**DOI:** 10.1017/S000711452200335X

**Published:** 2023-08-14

**Authors:** Katelyn M. Miller, Manju B. Reddy, David Quashie, Frank J. Velez, Jamel Ali, Prashant Singh, Stephen R. Hennigar

**Affiliations:** 1 Department of Nutrition and Integrative Physiology, Florida State University, Tallahassee, FL, USA; 2 Department of Food Science and Human Nutrition, Iowa State University, Ames, IA, USA; 3 Department of Chemical and Biomedical Engineering, FAMU-FSU College of Engineering, Tallahassee, FL, USA; 4 Pennington Biomedical Research Center, Baton Rouge, LA, USA

**Keywords:** Iron deficiency, Anaemia, Infection, Iron supplementation, Iron fortification

## Abstract

Excess unabsorbed iron in the gastrointestinal tract may select for enteric pathogens and increase the incidence and severity of infectious disease. *Aspergillus oryzae* (*Ao*) is a filamentous fungus that has the ability to accumulate and store large amounts of iron, and when used as a supplement or fortificant, has similar absorption to ferrous sulphate (FeSO_4_) in humans. The objective of this study was to determine the effect of iron-enriched *Ao* (*Ao* iron) compared with FeSO_4_ on iron accumulation, growth and motility of the Gram-negative enteric pathogen, *S.* Typhimurium. *S.* Typhimurium was cultured in media containing no added iron or 1 μM elemental iron as either *Ao* iron or FeSO_4_. *S.* Typhimurium cultured with FeSO_4_ accumulated more iron than those cultured with *Ao* iron. Genes regulated by the iron-activated transcriptional repressor, Fur, did not differ between control and *Ao* iron, but decreased in *S.* Typhimurium cultured with FeSO_4_ compared with both groups. Growth of *S.* Typhimurium was greater when cultured with FeSO_4_ compared with *Ao* iron and control. *S.* Typhimurium swam faster, had greater acceleration and travelled further when cultured with FeSO_4_ compared with *Ao* iron and control; swim speed, acceleration and distance travelled did not differ between *Ao* iron and control. These findings provide evidence that *Ao* iron reduces the virulence of a common enteric pathogen *in vitro*. Further research is required to determine whether iron-enriched *Ao* is a suitable iron supplement to improve iron delivery in areas with a high infection burden.

The World Health Organization recommends daily iron supplementation for infants and children (6 months – 12 years) and for menstruating, non-pregnant females, particularly in settings where the prevalence of anaemia is greater than 40 %^(1,2)^. Although universal iron supplementation is an effective approach to prevent iron deficiency and iron deficiency anaemia, safety concerns have been raised with these recommendations in areas with a high infection burden^(3)^. Based on the low cost and high bioavailability and efficacy, iron sulphate (FeSO_4_) is typically the first choice for supplementation and fortification; however, other inorganic iron sources are also commonly used^(4)^. The recommended dose of iron is set high to deliver adequate absorbed iron due to the low rate of dietary iron absorption^(5)^, which is typically <10 %^(6)^. Thus, the majority of dietary iron is not absorbed and travels to the colon. Unabsorbed iron in the colon may select for enteric pathogens at the expense of beneficial commensal bacteria and increase infection risk, including the clinical incidence of diarrhoea^(5,7–9)^.

Acquisition of iron is important for the proliferation and virulence of most enteric Gram-negative bacteria (e.g. *Salmonella*, *Shigella*, pathogenic *Escherichia coli* and *Campylobacter jejuni*). Enteric pathogens employ several mechanisms to scavenge iron from the host, such as synthesising iron-chelating siderophores that bind iron with high affinity, and through various iron uptake and efflux systems. One of the main ways pathogens regulate iron homeostasis is through transcriptional regulation of genes mediated by the ferric uptake regulator (Fur)^(10)^. When intracellular iron concentrations are high, the Fur protein acts as a transcriptional repressor by binding to specific sequences in the promoter and preventing transcription of Fur-regulated genes (e.g. *ton*B, *feo*B, *fim*A and *inv*A). In iron deplete conditions, Fur dissociates from the Fur box in the promoter and allows transcription of Fur-regulated genes to overcome iron limitation. *In vitro* studies consistently show greater proliferation of Gram-negative bacteria when cultured with inorganic iron sources^(11,12)^. Preclinical rodent studies have modelled an iron-restricted and iron-rich environment in the lower intestine by feeding varying levels of iron prior to oral inoculation with an enteric pathogen^(9,13)^. These studies have demonstrated iron-induced shifts in the gut microbiota, greater growth of enteric pathogens and a more severe enteropathy in mice fed diets fortified with FeSO_4_ compared with a low iron diet^(9,13)^. A randomised controlled trial found that iron-containing micronutrient powder provided to 6-month-old Kenyan infants adversely affected the gut microbiome and increased pathogen abundance^(7,8)^. These findings suggest the need for new approaches and alternative iron sources to correct or prevent iron deficiency and iron deficiency anaemia without increasing the risk or severity of infection.

*Aspergillus oryzae* (*Ao*) is a filamentous fungus that has the ability to accumulate and store large amounts of iron. Recent research has focused on *Ao* as a vehicle for iron supplementation and fortification^(14–17)^. In this application, *Ao* is grown in iron-rich media, harvested and ground into a fine powder. *Ao* grown in FeSO_4_ has been shown to have similar absorption to FeSO_4_ in women of reproductive age with low iron stores^(14)^. Bries *et al*. demonstrated that *Ao* iron is absorbed more slowly and produces less non-transferrin-bound iron 2–8 h post-supplementation compared with FeSO_4_^(16)^. In addition, the incidence of gastrointestinal side effects was lower after 3 weeks of supplementation with *Ao* iron compared with FeSO_4_. While studies have been conducted on the efficacy and safety of *Ao*, no study to date has determined whether *Ao* iron is a suitable alternative to inorganic iron in correcting or preventing iron deficiency without contributing to increased risk or severity of infection. The objective of this *in vitro* study was to determine the effect of *Ao* iron compared with FeSO_4_ alone on iron accumulation, growth and virulence of the Gram-negative enteric pathogen, *Salmonella enterica* subspecies enterica serovar Typhimurium (*S.* Typhimurium). *S.* Typhimurium is a rod-shaped, motile facultative intracellular pathogen whose greatest burden is in regions of the world where children are also affected by environmental enteric dysfunction^(18)^.

## Methods

### Strain and growth conditions

For all experiments, *S. enterica* subsp. enterica serovar Typhimurium ATCC® 14028™ was cultured in Iscove’s Modified Dulbecco Medium (IMDM; Quality Biological Inc.) at 37°C. IMDM contains no added iron. *S.* Typhimurium were cultured in media containing IMDM (control) or IMDM containing 1 μM elemental iron as either FeSO_4_ (BeanTown Chemical) or *Ao* grown with FeSO_4_ (Cura Global Health). This concentration represented the lowest dose of iron that produced the greatest difference in growth (online Supplementary Fig. 1) and is consistent with that used in previous studies^(11,12)^. The researchers were not blinded to the experimental treatments.

### Growth curves

*S.* Typhimurium were inoculated at 10^4^ CFU/ml. The concentration of inoculated *S.* Typhimurium was based on a previous study^(12)^ and optimised to maximise growth potential. *S.* Typhimurium were maintained in tryptic soy broth and subcultured twice in IMDM to limit residual iron from the tryptic soy broth. A sterile loop was used to transfer *S.* Typhimurium into a fresh culture tube containing 10 ml IMDM, vortexed and incubated overnight. The overnight culture was then vortexed, and 1 ml of overnight stock was transferred to a fresh tube containing 9 ml IMDM to create a 1:10 dilution of *S.* Typhimurium. IMDM alone (control) and IMDM containing FeSO_4_ or *Ao* iron were transferred into a sterile 15 ml conical tube (9·5 ml; *n* 6/treatment). Three tubes of each treatment were inoculated with 100 μl of *S.* Typhimurium using the above dilution; the other tubes were not inoculated with *S.* Typhimurium and served as blanks. To determine the effect of iron source on the growth of *S.* Typhimurium, 300 μl from each tube was pipetted into a sterile 96-well plate in triplicate. The 96-well plate was incubated at 37°C in a BioTek Synergy plate reader, and the optical density of the cultures was measured at 600 nm (OD_600_) every 30 min for 12 h. Plates were shaken before each reading.

### Iron accumulation

*S.* Typhimurium were cultured for 12 h as described above. Bacteria were pelleted, the pellet was rinsed to remove exofacially bound iron and bacteria were pelleted and digested in nitric acid. The iron concentration of the digested pellet was determined using flame atomic absorption spectrometry (Buck Scientific Instruments) and adjusted for OD_600_ to account for differences in bacterial growth. The iron content of IMDM without added iron was below the limit of detection (<0·01 mg/l).

### Real-time quantitative PCR (RT-qPRC)

Expression of Fur-regulated genes (tonB-dependent siderophore receptor, *ton*B-R; *ton*B system transport protein, *ton*B-TP; *feo*B; *fim*A; *inv*A) was measured by RT-qPCR. *S.* Typhimurium were cultured for 12 h as described above. Bacteria were pelleted, and RNA was isolated using TRIzol Reagent (Life Technologies). RNA quality and quantity were assessed using a NanoDrop One Spectrophotometer (Thermo Fisher Scientific). RNA was reverse transcribed with the High-Capacity cDNA Reverse Transcription Kit (Applied Biosystems) according to the manufacturer’s instructions. RT-PCR reaction was performed on a LightCycler 96 instrument (Roche Diagnostics Corp.) using 2x RT^2^ Green qPCR MasterMix (Qiagen). The RT-PCR amplification was performed in 10 µl reaction volume, using 0·15 µM of each primer (Table 1). The amplification consisted of an initial denaturation step of 900 s, followed by forty-five cycles of denaturation at 95°C for 15 s and annealing at 60°C for 30 s. All PCR amplification was performed with 20 ng of cDNA. Data were normalised to *16S*. The following equation was used to determine change in cycle quantification: ΔCq = Cq*Gene* – Cq*16S*. Fold change was calculated using the ΔΔCq method.

**Table 1.** Primer sequences

Primer	Sequence (5′-3′)
*ton*B-R-F	ACCTTCTTTGCTGCTTTCCG
*ton*B-R-R	GCCGGAGATGGTAGAACGTA
*ton*B-TP-F	CCTGAACCGCCTAAAGAAGC
*ton*B-TP-R	CGGCTGCTCTTCAACCTTTT
*feo*B-F	GCCGAAAATATTCAGGACGA
*feo*B-R	CTGCCGAATTTTTGATCCAT
*fim*A-F	GCAGGTGCCTTTCTCCATC
*fim*A-R	AGCGTATTGGTGCCTTCAAC
*inv*A-F	CGGTGGGTTTTGTTGTCTTC
*inv*A-R	TCATCGCACCGTCAAAGGA
16S-F	CCTCTTGCCATCGGATGT G
16S-R	GGCTGGTCATCCTCTCAGACC

### Motility

*S.* Typhimurium was cultured, pipetted into a 96-well plate and incubated at 37°C in a BioTek Synergy plate reader as described above. Optical density of the cultures was measured every 30 min until the point in the exponential growth phase where differences in growth between *S.* Typhimurium cultured in FeSO_4_ and *Ao* iron were greatest (∼7 h). Microscope slides were treated with 0·1 % TWEEN 20 (Sigma-Aldrich), dried and rinsed with ddH_2_O. *S.* Typhimurium (5 μl) was transferred from the 96-well plate to the slide (*n* 5 slides/treatment). A slide cover was placed on top of the slide, and the outer edges were sealed with nail polish to prevent airflow. Phase-contrast imaging was used to directly visualise bacterial motion in real time, and motion was recorded *via* a high-speed camera (*n* 5 videos/slide). The resulting videos were analysed using 2D tracking algorithms using NIS-Elements AR Analysis software (Nikon Instruments Inc.) to extract information such as velocity, acceleration and distance travelled of peritrichously flagellated *S.* Typhimurium. Particles tracked for less than 1 s were removed from the analysis and *S.* Typhimurium with a velocity greater than 3 μm/s were considered motile^(19)^.

### Statistics

Statistics were performed using GraphPad Prism, version 9.3.0. Data are presented as means ± SD for the number of samples reported. Experiments were repeated 2–3 times. Comparisons between iron source (control, FeSO_4_ or *Ao* iron) were performed using one-way ANOVA. Two-way ANOVA was used to determine the effects of iron source or concentration, time and their interaction. If a significant main effect was observed, *post hoc* comparisons were made using Tukey’s test. Data were tested for normality using Shapiro–Wilk’s test, and log-transformed data were used if data were not normally distributed. The *α* level for statistical significance was set at *P* < 0·05.

## Results

### Ao iron limits iron accumulation in S. Typhimurium compared with FeSO_4_

**Fig. 1.**
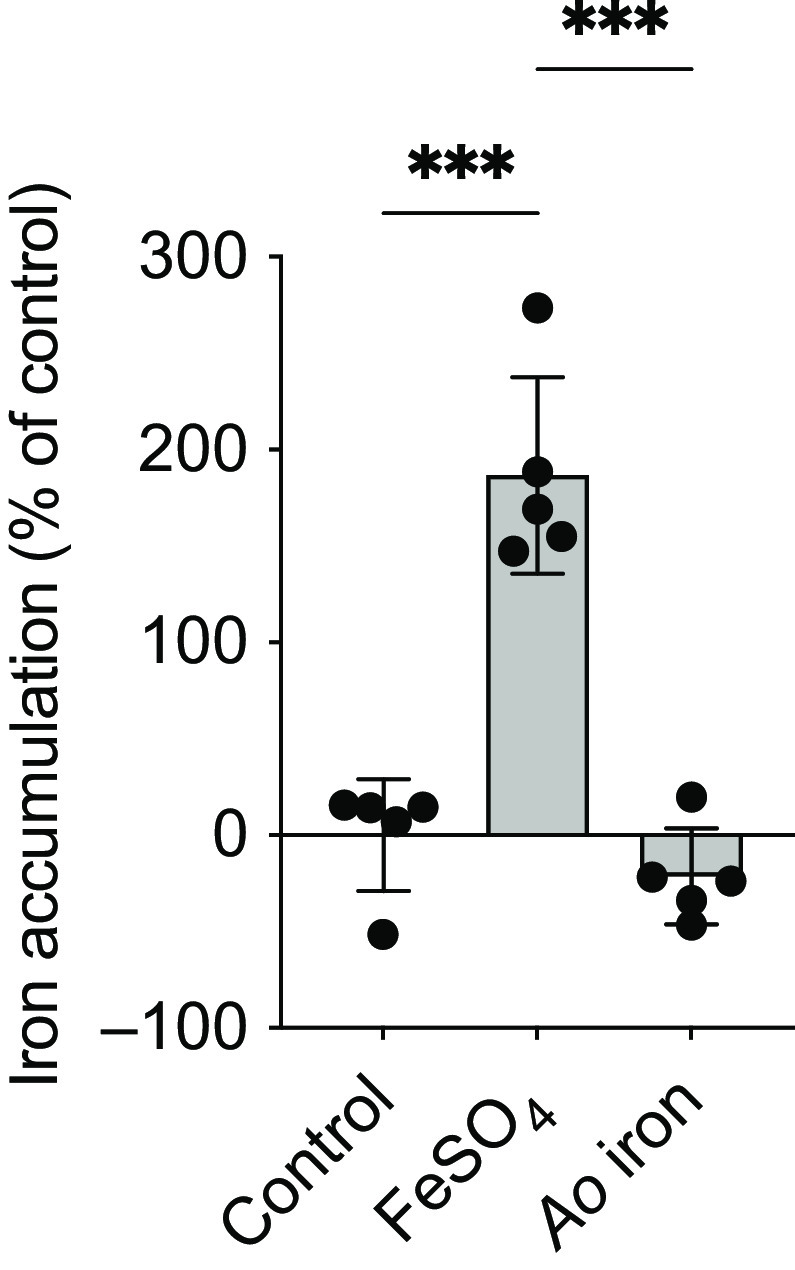
Iron accumulation is reduced in *S.* Typhimurium cultured with *Ao* iron compared with FeSO_4_. Iron accumulation in *S.* Typhimurium cultured in media containing no added iron (control) or 1 μM of elemental iron as either FeSO_4_ or *Ao* iron for 12 h. Data were analysed using a one-way ANOVA. Asterisks indicate a significant *post hoc* comparison (*P < 0·05; **P < 0·01; ***P < 0·001). Data are means ± SD; *n* 5/treatment.

*S.* Typhimurium cultured with FeSO_4_ accumulated ∼200 % more iron than those cultured in control media (*P* < 0·0001) and with *Ao* iron (*P* < 0·0001; Fig. 1). Iron accumulation did not differ in *S.* Typhimurium cultured in control media and with *Ao* iron (*P* = 0·64).

### Fur-regulated genes are upregulated with Ao iron compared with FeSO_4_

**Fig. 2.**
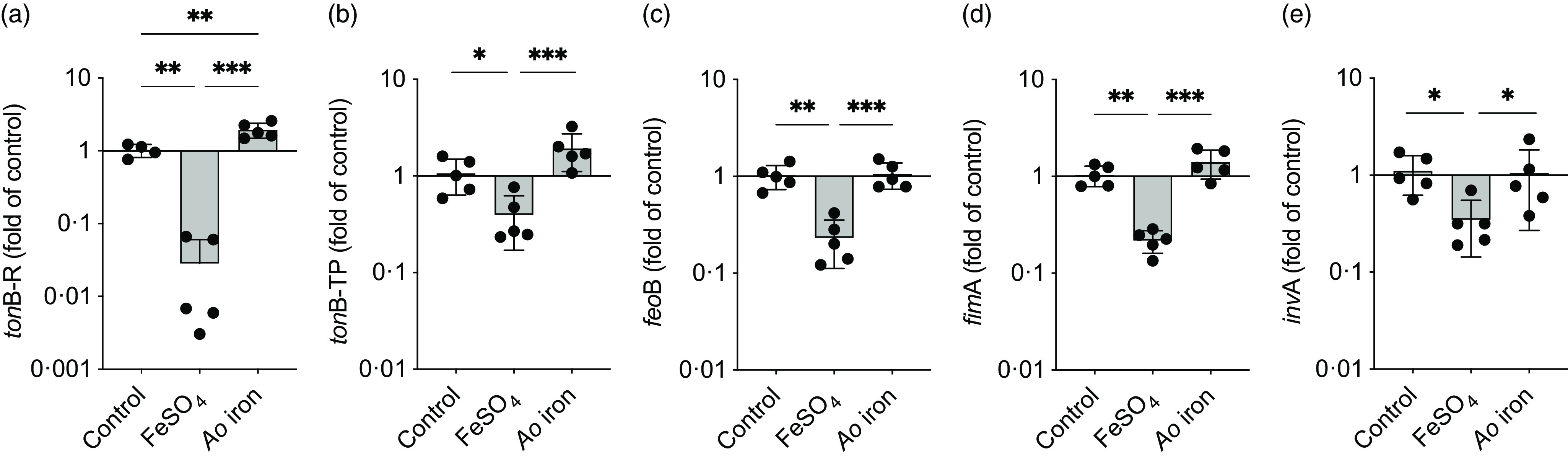
Fur-regulated genes are upregulated in *S.* Typhimurium cultured with *Ao* iron compared with FeSO_4_. Expression of: (a) *ton*B-R; (b) *ton*B-TP; (c) *feo*B; (d) *fim*A and (e) *inv*A in *S.* Typhimurium cultured in media containing no added iron (control) or 1 μM of elemental iron as either FeSO_4_ or *Ao* iron for 12 h. Data were analysed using a one-way ANOVA. *ton*B-TP and *inv*A were log transformed prior to analysis. Asterisks indicate a significant *post hoc* comparison (*P < 0·05; **P < 0·01; ***P < 0·001). Data are means ± SD; *n* 5/treatment.

Consistent with decreased iron accumulation in *S.* Typhimurium cultured with *Ao* iron, genes regulated by the iron-activated transcriptional repressor, Fur, were upregulated with *Ao* iron compared with FeSO_4_. *ton*B-R increased ∼1-fold with *Ao* iron compared with controls (*P* < 0·001) and decreased 48- and 26-fold with FeSO_4_ compared with *Ao* iron (*P* < 0·0001) and control (*P* < 0·0001), respectively (Fig. 2(a)). *ton*B-TP did not differ between *Ao* iron and controls (*P* = 0·88), but decreased ∼3- and 5-fold with FeSO_4_ compared with *Ao* iron (*P* = 0·02) and control (*P* = 0·01), respectively (Fig. 2(b)). *feo*B did not differ between *Ao* iron and controls (*P* = 0·99), but decreased ∼5-fold with FeSO_4_ compared with *Ao* iron (*P* < 0·0001) and control (*P* < 0·0001; Fig. 2(c)). *fim*A decreased ∼5- and 7-fold in *S.* Typhimurium cultured with FeSO_4_ compared with control (*P* < 0·0001) and *Ao* iron (*P* < 0·0001), respectively; controls and *Ao* iron did not differ (*P* = 0·42; Fig. 2(d)). *inv*A did not differ between *S.* Typhimurium cultured in control media compared with *Ao* iron (*P* = 0·88; Fig. 2(e)). There was a 3-fold reduction in *inv*A with FeSO_4_ compared with both *Ao* iron (*P* = 0·02) and control (*P* = 0·01).

### Ao iron restricts the growth of S. Typhimurium compared with FeSO_4_

**Fig. 3.**
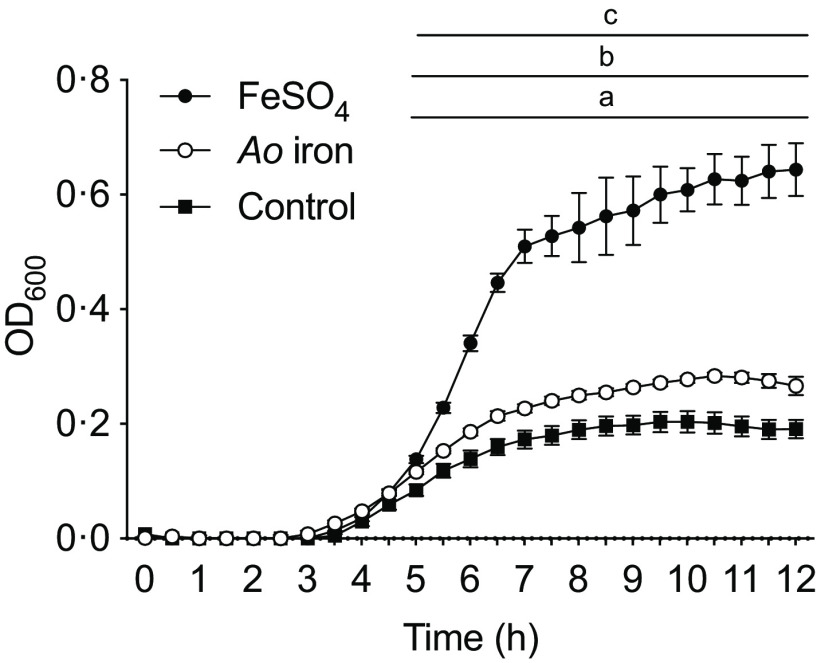
*Ao* iron restricts the growth of *S.* Typhimurium compared with FeSO_4_. Growth of *S.* Typhimurium cultured in media containing no added iron (control) or 1 μM of elemental iron as either FeSO_4_ or *Ao* iron. Data were analysed using a two-way ANOVA; main effects: *P*_time_<0·0001, *P*_treatment_<0·0001, *P*_interaction_<0·0001. Letters indicate a significant *post hoc* comparison (*P* < 0·05): (a) *P* < 0·05 FeSO_4_ compared with control; (b) *P* < 0·05 *Ao* iron compared with control; (c) *P* < 0·05 FeSO_4_ compared with *Ao* iron. Data are means ± SD; *n* 6/treatment/timepoint.

Growth of *S.* Typhimurium was greater in the presence of FeSO_4_ compared with controls (*P* < 0·0001), whereas growth was reduced in the presence of *Ao* iron compared with FeSO_4_ (*P* < 0·0001; Fig. 3). Time to reach peak doubling time was also greater with FeSO_4_ (5·5 ± 0·0 h) than *Ao* iron (4·6 ± 0·3 h; *P* = 0·0001) and control (4·5 ± 0·4 h; *P* < 0·0001), but did not differ between *Ao* iron and control (*P* = 0·96). Doubling time during the exponential growth phase was shorter with FeSO_4_ (35·5 ± 2·6 min) compared with *Ao* iron (57·3 ± 3·9 min; *P* < 0·0001) and control (49·5 ± 8·4 min; *P* = 0·001). Doubling time during the exponential growth phase did not differ between *Ao* iron and control (*P* = 0·06).

### Ao iron reduces motility of S. Typhimurium compared with FeSO_4_

**Fig. 4.**
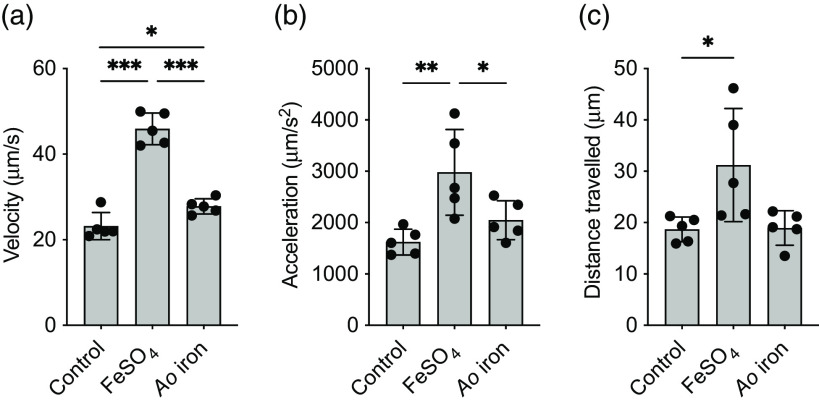
*Ao* iron restricts the motility of *S.* Typhimurium compared with FeSO_4_. (a) Velocity; (b) acceleration; and (c) distance travelled of *S.* Typhimurium cultured in media containing no iron (control) or 1 μM of elemental iron as either FeSO_4_ or *Ao* iron for 7 h. Data were log transformed prior to analysis and analysed using a one-way ANOVA. Asterisks indicate a significant *post hoc* comparison (*P < 0·05; **P < 0·01; ***P < 0·001). Data are means ± SD; *n* 5/treatment.

The velocity of *S.* Typhimurium cultured with *Ao* iron was not different from controls (*P* = 0·07), whereas *S.* Typhimurium cultured with FeSO_4_ swam 98 % faster than controls (*P* < 0·0001; Fig. 4(a)). Acceleration and the distance travelled followed a similar trend. Acceleration was similar between controls and *Ao* iron (*P* = 0·46; Fig. 4(b)), but increased by 84 % in *S.* Typhimurium cultured with FeSO_4_ compared with control (*P* < 0·01). Controls and *S.* Typhimurium grown in *Ao* iron travelled a similar distance (*P* = 0·99), whereas *S.* Typhimurium travelled approximately 65 % further when cultured in FeSO_4_ compared with control (*P* = 0·03; Fig. 4(c)).

## Discussion

During the course of infection, enteric pathogens compete with the host to obtain essential nutrients, such as iron. The current study shows for the first time that *Ao* iron attenuates iron accumulation, growth and motility of the common enteric pathogen *S.* Typhimurium compared with FeSO_4_ alone. The current study also offers further evidence that enteric pathogens efficiently accumulate inorganic iron sources, such as FeSO_4_, which contributes to their growth and motility. As previous studies in human subjects have demonstrated that iron absorption is similar between *Ao* iron and FeSO_4_^(14)^, findings from this study provide preliminary *in vitro* evidence that iron-enriched *Ao* may be a suitable iron supplement to improve iron delivery in areas with a high infection burden.

Acquisition of iron is necessary for the growth, survival and virulence of Gram-negative enteric pathogens^(20)^. Multiple *in vitro* studies demonstrate increased growth of enteric pathogens in the presence of inorganic iron, such as ferric citrate and ferric chloride, whereas *ton*B^−^ and *iro*N^−^ *fep*A^−^ mutants with an iron uptake defect have reduced growth when presented with iron compared with wild types^(11,12)^. Findings from the current study are consistent with these findings in that greater iron accumulation and growth was observed in the presence of FeSO_4_ compared with control. Importantly, we extend these findings and demonstrate that iron accumulation and growth of *S.* Typhimurium are restricted with *Ao* iron. Bries *et al*. reported that >90 % of the iron taken up by *Ao* is found within the mycelia and propose that iron from the complex fungal matrix is slowly absorbed over a longer period of time^(16)^. Increased iron accumulation with FeSO_4_ compared with *Ao* iron was confirmed by the observation that Fur-regulated genes were repressed with FeSO_4_, but were similar to control or greater with *Ao* iron. Fur monitors intracellular iron levels and regulates the transcription of genes involved in iron acquisition and utilisation and other cellular functions^(10)^. For example, *ton*B interacts with *iro*N and energises transport of ferric iron–siderophore complexes into the periplasm of Gram-negative bacteria^(21,22)^, and *feo*AB is a ferrous iron transporter consisting of the inner membrane transporter *feo*B and the cytosolic protein *feo*A, which aids *feo*B activity^(23)^. Collectively, these findings suggest that iron within *Ao* is sequestered, perhaps in the vacuole, and potentially unavailable to enteric pathogens, and may provide a mechanism underlying the reduction in iron accumulation and growth with *Ao* iron.

The ability of enteric pathogens to be motile is critical for scavenging nutrients, such as iron, in a competitive environment. Motility also enables bacteria to reach the intestinal epithelium and adhere to and invade cells^(24,25)^. In the current study, measures of motility (velocity, acceleration and distance travelled) were greater in *S.* Typhimurium cultured with FeSO_4_ compared with control and *Ao* iron, which did not differ from one another. These findings are consistent to those of Bearson *et al.* (2010) who reported increased motility in *Salmonella* cultured in media supplemented with 80 μM ferric chloride^(26)^; however, we extend these findings to show that *Ao* iron restricts the motility of *S.* Typhimurium when compared with FeSO_4_.

The amount of iron provided in micronutrient powders and fortification regimens balances the need to prevent or correct iron deficiency while minimising the risk of enhanced bacterial growth and virulence. Findings from the current study indicate that iron-enriched *Ao* restricts iron accumulation and virulence of a Gram-negative enteric pathogen *in vitro*. The finding that growth and motility with *Ao* iron were more similar to controls cultured without iron than to those cultured with FeSO_4_ provide promise that *Ao* iron may be an effective alternative to FeSO_4_. However, there are limitations to our findings. First, the current study used *S.* Typhimurium as a model Gram-negative enteric pathogen. Whether *Ao* iron has similar effects on other Gram-negative pathogens remains to be determined. Similarly, examining iron accumulation, growth and motility in pathogens that do not require iron may provide further insight into the mechanism by which *Ao* iron is withheld. Findings from the current *in vitro* study also need to be validated in a more complex *in vivo* model. One important question is the nature of *Ao* iron after digestion. The slow release of iron into the blood and lower non-transferrin-bound iron with *Ao* iron compared with FeSO_4_^(16)^ suggests that the fungal matrix is slowly digested and that at least some iron is still sequestered in mycelia, but this has not been specifically tested. These and other questions are important for determining whether iron-enriched Ao is a suitable supplement or fortificant to improve iron delivery in areas with a high infection burden.

## Supplementary material

For supplementary material accompanying this paper visit https://doi.org/10.1017/S000711452200335X. click here to view supplementary material
