# Supplementary material for: Iron-enriched Aspergillus oryzae as an alternative to iron sulphate to limit iron accumulation, growth and motility of the enteric pathogen S. Typhimurium
Source: Br J Nutr. 2022 Oct 20;130(3):411–6. doi: 10.1017/S000711452200335X (PMC10331431; doi:10.1017/S000711452200335X)
Supplement: Supplementary file 1 [file S000711452200335Xsup001.docx]

**Supplementary Figure 1. Growth of *S.* Typhimurium in media with varying concentrations of FeSO_4_ and *Ao* iron.** *S.* Typhimurium was cultured in IMDM containing no iron (control) or 0.1, 1, or 5 μM elemental iron as either FeSO_4_ or *Ao* iron. Data are means ± standard deviations; n=6/treatment/timepoint.
